# Verbal emotional memory laterality effect on amygdalohippocampectomy for refractory epilepsy

**DOI:** 10.1002/brb3.1872

**Published:** 2020-10-05

**Authors:** Fernando Carvajal, Ainara Calahorra‐Romillo, Sandra Rubio, Pilar Martín

**Affiliations:** ^1^ Biological and Health Psychology Department Universidad Autónoma de Madrid Madrid Spain

**Keywords:** amygdalohippocampectomy, emotion, epilepsy, episodic memory, memory, neurosurgery, verbal emotional memory

## Abstract

**Objectives:**

To study the brain lateralization of the verbal emotional memory and the influence of the emotional valence, we investigated a sample composed of patients with medial temporal lobe refractory epilepsy (MTLE) treated with unilateral amygdalohippocampectomy compared to a control group.

**Materials & Methods:**

A new task (Verbal Association) was designed and implemented to assess emotional memory performance. It was applied to 62 patients with MTLE of whom 31 have been subjected to right amygdalohippocampectomy and 31 to left amygdalohippocampectomy. These patients were compared with 31 participants with no cerebral pathology, as a control group.

**Results:**

(a) The control group obtained a higher number of recalled words than the rest of the groups, while the MTLE‐right group obtained better results than the MTLE‐left group. (b) In the case of positive emotional valence words, the MTLE‐left group performed significantly worse than the rest of the groups; whereas for negative emotional words, the MTLE‐left group presented the lowest average performance and the control group obtained a higher number of recalled words compared to MTLE‐right group. In the case of neutral emotional words, no significant differences were found among the groups. (c) The MTLE‐left group showed poorer performance on positive and negative words than neutral; the control group demonstrated lower average performance on positive and neutral words compared to negative; the MTLE‐right group did not show any significant differences on the recall of different emotional valences.

**Conclusions:**

Patients with MTLE show a deficit in the verbal recall which is exacerbated for information with an affective component. This deficit is more prominent in the case of patients with left unilateral resection (MTLE‐left group) since they lose the benefits of the emotional information for the recall.

## Graphical abstract

We studied the lateralization of verbal emotional memory depending on the emotional valence using a sample composed of subjects with medial temporal lobe epilepsy (MTLE) who received an amygdalohippocampectomy and a control group. Patients with MTLE show a deficit in the verbal recall which is exacerbated for information with an affective component. This deficit is more prominent in the case of patients with left unilateral resection (MTLE‐left group) since they lose the benefits of the emotional information for the recall.

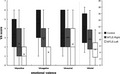

## 1 INTRODUCTION

Emotional Episodic Memory might be defined as the capacity of encoding, storing, and retrieving emotional information, so that, highly emotional events are better recalled than those that are not significantly affective (Adelman & Estes, 2013; Buchanan, 2007; Kensinger & Corkin, 2003; Wang, 2020). People presenting difficulties in the retrieval of emotional memories show some limitations while managing affective events, which may influence their social, family, or work relationships (Carcone & Ruocco, 2017; Walla & Panksepp, 2013; Wilhelm et al., 2014).

Although it is widely accepted that the emotional information increases the recall response, a process in which the hippocampus and the amygdala´s circuits are implicated, it is not clear what the influence of the emotional valence is (positive, negative or neutral value), or what the differences between the hemispheres are (Bennion et al., 2013; Kennepohl et al., 2007; Langston et al., 2010; Rice et al., 2018). In this regard and based on the studies conducted with patients with left or right unilateral cerebral damage, there is an agreement relating the negative emotions with the right hemisphere and the positive ones in the left (Johansson et al., 2004; Rodway et al., 2003; Roesmann et al., 2019; Sergerie et al., 2005; Wyczesany et al., 2018). In contrast, other studies found specialization for the negative emotions and not for the positives ones (Adolphs et al., 1995; Burton & Labar, 1999; Frank & Tomaz, 2003; Jambaque et al., 2009; Pinabiaux et al., 2013). Some studies even found a strong relationship between negative emotions and the left cerebral hemisphere (Beraha et al., 2012; Mneimne et al., 2010).

The knowledge of the cerebral bases of the emotional memory can particularly benefit from the study of patients with mesial temporal lobe epilepsy (MTLE) who have been subjected to unilateral resection of the hippocampus and amygdala. These patients usually present some difficulties in emotional facial recognition (Adolphs et al., 2001; Carvajal et al., 2007, 2009) and especially on the episodic memory. There is an agreement on findings that difficulties in the recall of verbal information are the ones that most restrict the patients. Besides, these difficulties in remembering verbal information are more remarkable in patients that have been subjected to left unilateral resection (Martin et al., 2002; Schwarcz & Witter, 2002; Tudesco et al., 2010). Therefore, this work aims to address the study of the emotional memory under verbal stimuli with positive, negative, and neutral valence. To do that, the verbal emotional memory was studied by a sample of patients with unilateral temporal lobectomy (right or left) of the hippocampus and amygdala region. These patients were compared with participants with no cerebral pathology as control. Emotional memory deficits are not commonly explored after surgery, and understanding the relationship between surgery and behavior is necessary not only for better postsurgical management but also for a better presurgical selection.

In this study, we hypothesized that healthy participants with no cerebral pathology could recall more verbal information than patients with cerebral pathology. We also wanted to confirm the hypothesis that those patients with left temporal lobectomy could have the lowest performance on remembering verbal information. Then, regarding the influence of the emotional valence in the recall of verbal information, we hypothesized that patients with right temporal lobectomy presented higher impairment on negative verbal information recall, whereas patients with left temporal lobectomy had more difficulties on positive verbal information recall.

## 2 MATERIAL AND METHODS

### 2.1 Participants

We followed the protocol from the Neurosurgery Service of the Hospital Universitario de la Princesa (Madrid, Spain), which implies neurology, psychiatry, neurophysiology, imaging, and neuropsychology. In particular, for neuropsychology, we used the Wechsler Adult Intelligence Scale, Third Edition (WAIS‐III) (Wechsler, 2001), and the Logical Memory I (LM‐I) of the Wechsler Memory Scale‐Revised (WMS‐R) (Wechsler, 2004) were administered to the participants. Then, we applied as exclusion criteria: (a) having an IQ below 70; (b) not having completed their primary education; (c) having previous neurological pathology different to epilepsy; (d) having psychiatric disorders according to the Diagnostic and Statistical Manual of Mental Disorders IV‐TR (DSM‐IV‐TR) (American Psychiatric Association, 2004), or (e) not having completed the assessment. Ninety‐three participants (45 males and 48 females) were finally selected for this study.

All of the participants signed the informed consent form and were divided into three groups, 31 patients with MTLE and right temporal lobe amygdalohippocampectomy (MTLE‐right group), 31 patients with MTLE and left temporal lobe amygdalohippocampectomy (MTLE‐left group), and 31 participants with no cerebral pathology (control group). The control group was composed of family members of the patients.

The surgery of the patients from groups MTLE‐right and MTLE‐left consisted of cortical resection and selective amygdalohippocampectomy through the Spencer Technique (Spencer & Ojemann, 1993).

The entire patient's sample of the study (MTLE‐right and MTLE‐left groups) was included in the epilepsy surgery protocol due to failure to satisfactorily respond to pharmacological treatment. The study was conducted in the Epilepsy Surgery Unit. The lobectomy included resection of 3 cm from the tip of the temporal lobe, the head, and the anterior third of the hippocampus, the uncus, and the entire amygdala. Postsurgery assessment was done from 6 to 8 months after the clinical intervention.

As can be noticed in Table 1, the three groups matched on age, sex, educational level, employment situation, and general intellectual performance (IQ from WAIS‐III). IQ from all the sample ranged from 82 to 126, and all participants were right‐handed as assessed with the Edinburgh Handedness Inventory (Oldfield, 1971) (EHI > 80). The analysis of the verbal episodic memory (LM‐I from WMS‐R) revealed a statistically significant difference across the groups, (*F* (3, 116) = 25.94; *p* < .001). The control group recalled more information than the other two groups of patients (Tukey a *p_s_* < .001), and the MTLE‐left group obtained a lower average performance recalling verbal information than MTLE‐right (Tukey a *p* < .01).

**Table 1:** Average values and standard deviations (*SD*) of age, general cognitive ability (IQ) and verbal episodic recall (LM‐I), and absolute numbers and percentage of sex, educational level, and employment situation from all participants in the sample of the study

	MTLE‐right (*n* = 31)		MTLE‐left (*n* = 31)		control (*n* = 31)		*p*‐value
	Average	*SD*	Average	*SD*	Average	*SD*	
Age	35.14	11.61	37.35	10.14	37.48	10.51	.064
IQ	101.39	15.51	94.69	14.58	98.90	11.62	.170
LM‐I	30.56	26.05	14.97	20.29	64.03	26.09	.001^**^
Sex: *n* (%)							.14
Male	15 (48.4%)		17 (54.8%)		13 (41.9%)		
Female	16 (51.6%)		14 (45.2%)		18 (58.1%)		
Educational level: *n* (%)							.14
Primary	16 (51.6%)		17 (54.8%)		15 (48.4%)		
Secondary	9 (29.0%)		9 (29.0%)		7 (22.6%)		
Higher	6 (19.4%)		5 (16.1%)		9 (29.0%)		
Employment: *n* (%)							.15
Unemployed	16 (51.6%)		15 (48.4%)		12 (38.7%)		
Active	15 (48.4%)		16 (51.6%)		19 (61.3%)		

Note

The values on the *p*‐value column correspond to the *p*‐value obtained from a one‐factor ANOVA test (for age), a chi‐squared test (for sex), a Kruskal–Wallis test (for educational level and employment) and a repeated measures ANOVA with Group as a factor (for LM‐I).

^**^ *p*‐value is <=.01.

Participants were informed of the confidential and anonymous treatment of their data and signed the informed consent. The study was completed following the Helsinki Declaration and was approved by the Ethical Committee of the Universidad Autónoma de Madrid.

### 2.2 Instruments and procedures

The study was performed during two sessions of 1 hr each. In the first session, we assessed general cognitive ability using the Wechsler Adult Intelligence Scale (WAIS‐III) and the performance of the verbal episodic memory using the Logical Memory (LM) of Wechsler Memory Scale‐Reviewed (WMS‐R). During the second session, we assessed the Emotional Memory by a new task designed for this study denominated Verbal Association (VA).

The VA is composed of a list of pairs of words, in which the first one of each pair is the signal and the second is the target that participants should remember. In this study, the signal word is always neutral (no emotional content) and the target word can be either positive, negative, or neutral. We chose this task because its performance depends on the long‐term memory even when the retention interval is brief (Kim et al., 2011). The main advantage of utilizing this task with supraspan material is that the words can be presented independently with different emotional valence.

The words composing this task were selected from other studies in Spanish (Blanch & Baños Rivera, 1996; Jambaque et al., 2009; Redondo et al., 2005). Other additional words were added to the preliminary list, taking into account the length and frequency of use (Real Academia Española, 2018). This preliminary list of words was subjected to inter‐rater reliability through 100 bachelor students to confidently assign each of them to one of the three emotional valences (positive, negative, or neutral). The final list was composed of 12 words per category, being the ones with the best consensus among the inter‐rater reliability task.

**FIGURE 1:**
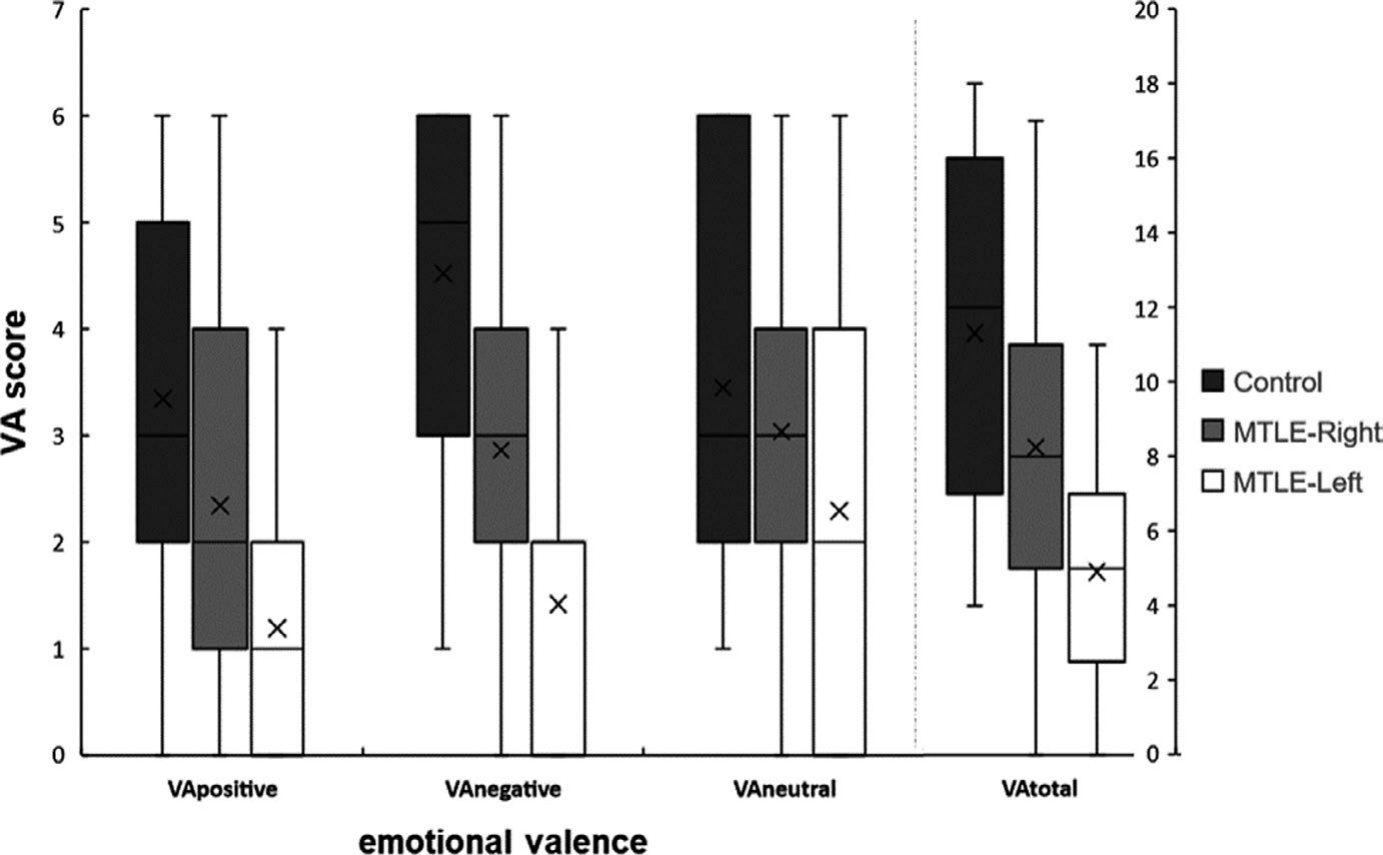
Box and Whisker plot summarizing the distribution of scores obtained from the Verbal Association task distinguishing by groups and emotional memory. The right vertical axis only corresponds to the VAtotal score

The VA task was composed of 6 target words structured in 6 pairs of words. The selected target words were two positives: *premio* (*award*) and *chiste* (*joke*), two negatives: *guerra* (*war*) and *muerte* (*death*), and two neutrals: *sello* (*stamp)* and *casa* (*house*). This task was composed of three assays, performed with no pause between them. In each one, the clinician read the list of pairs of words, and subsequently, the clinician named, in a different order, the signal word of each pair, while the participant attempted to answer the target word associated with it without the pressure of time. Participants did not receive feedback about the outcome per essay. In the analysis of the recalled words, participants obtained four scores: *VAtotal* or total number of words recalled (maximum 18 words); *VApositive* or number of words recalled with positive valence (maximum 6 words), *VAnegative* or number of words recalled with negative valence (maximum 6 words), and *VAneutral* or number of words recalled with neutral valence (maximum 6 words). Figure 1 shows the distributions of these scores obtained by the three groups.

In a first pilot study, we used a task composed of 9 pairs of words, with the idea of avoiding any ceiling effect; however, this task resulted to be too complex for most of the participants who, for that reason, frequently were not able to complete it. Therefore, we opted for the current 6 pairs of words version of the task.

## 3 RESULTS

### 3.1 Verbal Association without emotional valence

First, we statistically analyzed differences among the groups in the amount of information that the participants were able to remember during the three codification assays, regardless of their emotional valence, that is, attending to the scores *VAtotal* (right side of Figure 1). A single‐factor ANOVA was conducted where the dependent variable was the total number of words recalled. The analysis displayed a significative effect of group factor (*F* (2, 92) = 19.17, *p* < .001); and the posterior contrast with a post hoc Tukey test indicated that the control group recalled significantly more words than the two patient groups (Tukey *p_s_* < .05 and 0.001, MTLE‐right group and MTLE‐left group, respectively). At the same time, the MTLE‐left group obtained significantly worst results than the MTLE‐right group (Tukey_a_ *p* < .01).

### 3.2 Verbal Association with emotional valence

In the second set of analyses, we focused on the emotional valence using the scores *VApositive*, *VAnegative*, and *VAneutral*, which correspond to the number of positive, negative, and neutral words correctly remembered by the participants during the three codification assays, respectively. Figure 1 shows the distributions of the scores across groups and emotional valences.

A two factors group × emotional valence ANOVA of repeated measures was computed (with emotional valence as repeated factor) on the amount of information that the participants were able to remember during the three codification assays. This analysis revealed a main effect for the emotional valence (*F*(2, 180) = 6.74, *p* < .01) indicating that the recall of positive words was lower than the recall of negative and neutral words (Tukey_a_, *p_s_* < .01 and .05), not finding significant differences between these two (Tukey_a_, *p* = .75).

The effect of group variable was also significant (*F*(2, 90) = 19.32, *p* < .0001), indicating that the control group obtained a higher number of recalled words than the patient's groups (Tukey_a_, *p_s_* < .01). MTLE‐right group obtained better results than the MTLE‐left group (Tukey_a_, *p* < .05).

Finally, the interaction between the two factors was also significant (*F*(4, 180) = 6.08, *p* < .01). Later analyses revealed a significative effect on the recall of positive (*F*(2, 90) = 12.14, *p* < .001) and negative words (*F*(2, 90) = 35.01, *p* < .001) but not in the case of the neutral words (*F*(2, 90) = 2.57, *p* = .08). For words with positive emotional valence, MTLE‐left group significantly recalled fewer words than the other groups (Tukey_a_, *p_s_* < .01 and .05), and the performances of control and MTLE‐right groups were similar (Tukey_a_, *p* = .12). Once again, in the case of negative emotional words, the MTLE‐left group presented the lowest average performance (Tukey_a_, *p_s_* < .01). Likewise, the control group obtained a higher number of recalled words than the MTLE‐right group (Tukey_a_, *p* < .01).

In addition, we independently analyzed each group to detect significant differences in the performance of the recall depending on the emotional valence by performing an ANOVA of repeated measures with emotional valence as repeated factor. Its results revealed indicative differences among the emotional valences in the MTLE‐left group (*F*(2, 60) = 6.04, *p* < .01) and the control group (*F*(2, 60) = 10.27, *p* < .001), but not in the MTLE‐right group (*F*(2, 60) = 2.19, *p* = .12). The MTLE‐left group showed poorer performance on positive and negative words than neutral (Tukey_a_, *p_s_* < .05 and .01) and no differences between the recall of positive and negative words (Tukey_a_, *p* = .35). The control group demonstrated lower average performance on positive and neutral words than negatives (Tukey_a_, *p_s_* < .01), with no differences between the recall of positive and neutral valence (Tukey_a_, *p* = .84).

## 4 DISCUSSION

Data from three groups of participants were collected to perform this study. The results obtained in the analysis of the emotional verbal association indicated that, as occurred in the task of verbal recall (LM‐I) on WMS‐R, the patients with MTLE obtained lower scores than the control participants. Also, left unilateral resection is associated with higher difficulties than the right unilateral resection. This result matches with previous studies and highlights the preponderating left hemisphere over the right in the verbal recall (Bell & Giovagnoli, 2007; Kennepohl et al., 2007; Martin et al., 2002). Additionally, the results of this study indicated that the three groups did not differ in the recall of neutral words; hence, the deficit on verbal memory presented by the MTLE patients is limited to the emotional words since we found significant differences in the recall of positive and negative emotional words.

We also observed that the emotional valence exhibits a differential weight in participants with amygdalohippocampectomy (MTLE) compared to participants without cerebral pathology. We did not observe significant differences in the recall of emotional words with positive valence compared to words with negative valence in participants with amygdalohippocampectomy, whereas participants from the control obtained a significantly better performance on the recall of emotional words with negative valence compared to positive valence ones.

Moreover, the patients with surgical resection present a distinguishing effect depending on which hemisphere was intervened. This effect coincides with other authors in the case of right unilateral resection (Adelman & Estes, 2013; Hermans et al., 2014; LeDoux, 2003, 2012; Rice et al., 2018; Roesmann et al., 2019; Wyczesany et al., 2018), indicating that the negative valence of verbal information does not influence the retrieval. However, the hypothesis postulating that left unilateral resection should affect the recall of positive information is not confirmed. This means that patients with left unilateral resection lose the benefits in the recall of emotional information as it happens to patients with right unilateral resection, and however, patients with left unilateral resection present additional difficulties in the recall of emotional verbal information, being so that they recall emotional words (positives or negatives) worse than neutral words. Therefore, in this case, emotional information becomes an impediment of the recall, producing a counter‐productive effect on patients with left surgery.

Taking the results altogether, we conclude that patients with MTLE show a deficit in verbal memory which is more prominent when the information has an affective component. Moreover, this deficit is especially clear for patients with left unilateral resection, who recall less information than the rest and who additionally lose the benefit of recalling emotional information.

In this study, we have wanted to focus solely on the verbal emotional memory. In the future, we will also study the observation of other types of memory, such as the visual emotional memory, which could also yield additional information especially in the case of our MTLE‐right group of patients, for whom verbal memory could be affected by the temporal lobe resection.

Many previous studies demonstrated that patients with MTL damage (regardless of surgical removal) often present difficulties in emotional memory (Adolphs et al., 2000; Buchanan, 2007; LaBar & Phelps, 1998; Todorov & Olson, 2008). In this regard, not having information about the MTLE patients before the surgery is a limitation in our study that we could not circumvent.

Future studies could also address the investigation of whether the affectation on emotional memory may cause difficulties in cognitive and emotional functional regulation. In this sense, people that cannot conveniently recall emotional information could present limitations in the learning of affective events, which could influence their social, family, and labor relations (Wilhelm et al., 2014). This approach becomes especially relevant in the case of patients with left amygdalohippocampectomy who do not present the beneficial effect of emotional information to improve the recall. Not only that, but the emotional memory also interferes with the recall.

## CONFLICT OF INTEREST

Authors declare no competing financial interests with the work described.

## AUTHOR CONTRIBUTION

SR, FC, and ACR designed the new task, performed the task, and acquired the data. FC and ACR performed the statistical analysis of the data. ACR drafted the manuscript. ACR, FC, SR, and PM provided critical revision of the manuscript. PM conceived and supervised the project. All authors critically reviewed content and approved the final version for publication.

### Peer Review

The peer review history for this article is available at https://publons.com/publon/10.1002/brb3.1872.

## Data Availability

The data that support the findings of this study are available on request from the corresponding author. The data are not publicly available due to privacy or ethical restrictions.
